# Surface-Functionalized Polystyrene Nanoparticles Alter the Transmembrane Potential via Ion-Selective Pores Maintaining Global Bilayer Integrity

**DOI:** 10.1021/acs.langmuir.2c02487

**Published:** 2022-11-23

**Authors:** D. Aurora Perini, Elisa Parra-Ortiz, Inmaculada Varó, María Queralt-Martín, Martin Malmsten, Antonio Alcaraz

**Affiliations:** † Laboratory of Molecular Biophysics, Department of Physics, Universitat Jaume I, 12071Castellón, Spain; ‡ Department of Pharmacy, University of Copenhagen, DK-2100Copenhagen, Denmark; § Institute of Aquaculture Torre de la Sal (IATS-CSIC), Ribera de Cabanes, 12595Castellón, Spain; ∥ Department of Physical Chemistry 1, University of Lund, SE-22100Lund, Sweden

## Abstract

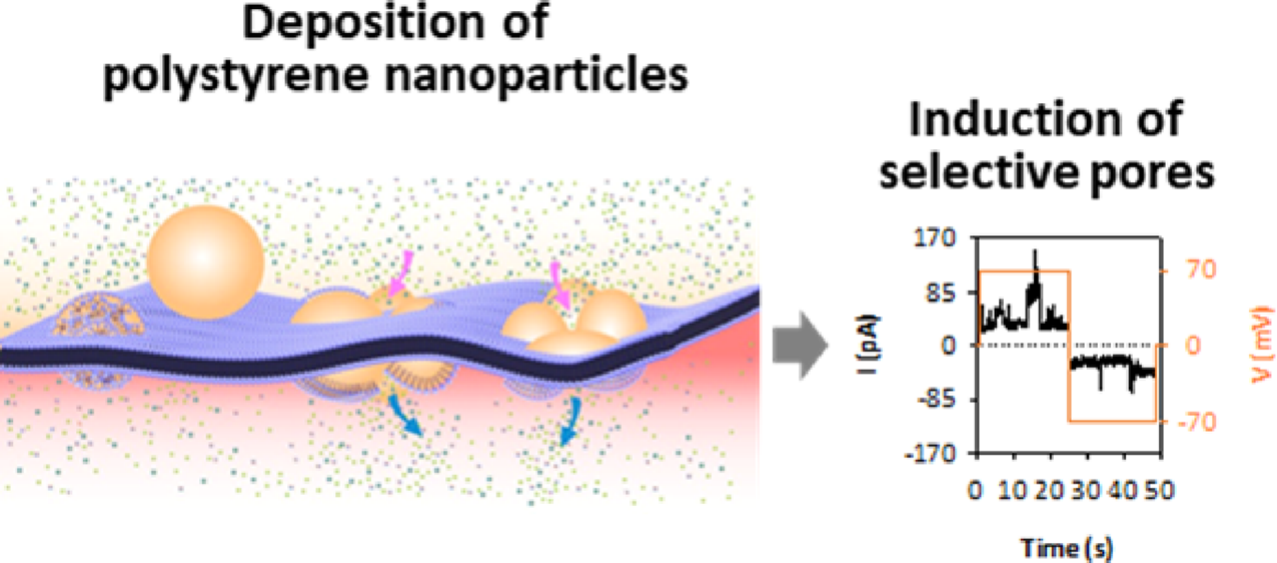

Although nanoplastics have well-known toxic effects toward the environment and living organisms, their molecular toxicity mechanisms, including the nature of nanoparticle–cell membrane interactions, are still under investigation. Here, we employ dynamic light scattering, quartz crystal microbalance with dissipation monitoring, and electrophysiology to investigate the interaction between polystyrene nanoparticles (PS NPs) and phospholipid membranes. Our results show that PS NPs adsorb onto lipid bilayers creating soft inhomogeneous films that include disordered defects. PS NPs form an integral part of the generated channels so that the surface functionalization and charge of the NP determine the pore conductive properties. The large difference in size between the NP diameter and the lipid bilayer thickness (∼60 vs ∼5 nm) suggests a particular and complex lipid–NP assembly that is able to maintain overall membrane integrity. In view of this, we suggest that NP-induced toxicity in cells could operate in more subtle ways than membrane disintegration, such as inducing lipid reorganization and transmembrane ionic fluxes that disrupt the membrane potential.

## Introduction

Polystyrene nanoparticles (PS NPs) are produced for a variety of technological applications. Primary uses of such particles include pharmaceuticals, cosmetics, paints, medical and electronic devices, plastic packaging, and construction materials.^1−5^ As a result of this, nanometer-sized PS is abundantly present in the environment due to accidental release of produced NPs^6,7^ but also due to degradation of bulk PS via mechanical breakdown,^8^ UV degradation,^9,10^ and/or biodegradation.^11^ Given that both micro- and nanoplastics have been detected mostly in a watery environment,^1^ ecotoxicological studies have paid special attention to aquatic species, demonstrating that PS NPs could accumulate in tissues, alter the immune system, produce cell damage, and/or induce oxidative stress and genotoxicity, which affect survival, growth, and reproduction.^4,12−17^

Cell membranes are the primary protective barrier of living organisms against toxic agents^8,18^ and particularly NPs.^19^ NP–membrane interactions involve a complex balance between elastic and adhesion forces that define the degree of membrane deformation and NP wrapping by lipids.^20^ Given their hydrophobic nature, noncross-linked plastic NPs could potentially dissolve in the hydrophobic core of bilayers^21^ as shown by molecular dynamics simulations for globular polyethylene NPs with a diameter of few nanometers. However, it is unclear whether this mechanism is viable for large NPs (*d* > 50 nm) exceeding by more than one order of magnitude the bilayer thickness. Possible scenarios for such large NPs include full NP engulfment (similar to nonspecific endocytosis), partial wrapping, NP adsorption to the membrane external surface,^20^ or disintegration of the NP into individual polymer chains.^22^

Interestingly, cell culture experiments show that cellular binding of NPs disrupts the membrane potential, not only altering the normal cell cycle progression but also inducing malignant transformations and avoiding tissue regeneration.^23^ In vitro techniques taking advantage of the fact that PS NPs can be adsorbed on model membranes^24−29^ have stimulated intense research providing molecular details not visible at the cellular level. Thus, the alteration of ionic gradients around cells by PS NPs has been linked to a variety of molecular mechanisms such as NP internalization,^30^ local collapse of membrane integrity,^25^ creation of hydrophilic pores,^31^ or even the physical blockage of ion channels such as K^+^ or Na^+^ ones.^23^ These mechanisms are not mutually exclusive but could operate in concert or even synergistically. For instance, in membranes where large NPs are present, pore formation can provide a stabilizing mechanism releasing membrane tension and avoiding global membrane rupture.^19^ Thus, the presence of defects or pores in the membrane could impact the membrane potential in two ways: on the one hand, allowing the unassisted transport of hydrophilic solutes and salt ions, and on the other, altering the passive lipid translocation (lipid flip-flop) required for mechanical stability and membrane protein activity.^32^

To gain insight into nanoplastic–membrane interactions, we combined experimental techniques in which 60 nm PS NPs with different surface functionalizations interact with membranes mimicking the phospholipid composition of those from endoplasmic reticulum, mitochondria, lysosome^33−35^ or the *Artemia franciscana* brine shrimp eyes,^36−38^ a model species particularly relevant in NP aquatic toxicology.^15,39−41^ Dynamic light scattering (DLS) was employed to provide colloidal characterization of the NPs in terms of ζ-potential, size, and polydispersity at biologically relevant pH and salinity. Afterward, the surface-sensitive technique quartz crystal microbalance with dissipation monitoring (QCM-D) was used to investigate the NP deposition in real time on supported lipid bilayers and subsequent changes in membrane viscoelasticity. Finally, electrophysiology experiments on planar lipid membranes assessed the membrane integrity upon addition of PS NPs and the potential occurrence of pore formation by monitoring conductivity changes. We observe that under physiological conditions, the studied NPs attach to the lipid surface and form disordered conductive pores that resemble proteolipid pores formed by association of membrane proteins and lipid molecules.^42−52^ By using a combination of different electrophysiological parameters, we elucidate the conductive nature of the observed pores, the participation of PS NPs in their structure, and the role of NP charge and functionalization. Based on this, we discuss the possible role of NP-induced pores and their role for membrane organization in nanoplastic toxicology.

Our systematic investigation on model biological membranes using a combination of techniques could be of much interest to elucidate the role of surface chemistry on the propensity of all engineered nanomaterials (both carbon-based and inorganic) to disrupt cell membranes, paving the way to design safer biocompatible particles suitable for environmental and biotechnological applications.^53^

## Experimental Section

### Materials

Three types of NPs (60 nm diameter) were purchased from Magsphere Inc. (Pasadena, CA) and included: nonfunctionalized polystyrene (PS) (plain NP according to manufacturer’s nomenclature), aminated polystyrene (PS-NH_2_), and carboxylated polystyrene (PS-COOH). Lipids dissolved in chloroform, 1,2-dioleoyl-*sn*-glycero-3-phosphocholine (DOPC), 1,2-dioleoyl-*sn*-glycero-3-phospho-l-serine (DOPS), and 1,2-dioleoyl-*sn*-glycero-3-phosphoethanolamine (DOPE) were purchased from Avanti Polar Lipids (Alabaster, AL). Sodium chloride and 4-(2-hydroxyethyl)-1-piperazineethanesulfonic acid (HEPES) were obtained from Merck KGaA (Darmstadt, Germany). All solutions were prepared using ultrapure water (UW) (Simplicity UV, Merck KGaA, Darmstadt, Germany) with a resistivity of 18.2 MΩ/cm at 25 °C and filtered through a 0.20 μm pore size filter (Scharlau, Spain).

### NP Characterization

PS NPs were analyzed at different salt concentrations by DLS using a Zetasizer Nano ZSP (Malvern Panalytical Ltd., Malvern, UK) at a scattering angle of 173° to characterize the hydrodynamic sizes and ζ-potentials. For size determination, three sets of measurements of 10 runs of 10 s were used, whereas ζ-potentials were determined based on 30 runs and monomodal analysis. Automatic attenuation was used for all samples. The ζ-potential values were calculated from the electrophoretic mobility by applying the Smoluchowski equation.^54^ Measurements were performed in triplicate at *T* = 25.0 ± 0.5 °C and reported as the mean ± standard deviation.

### Preparation of Liposomes

Small unilamellar vesicles (SUVs) for QCM-D were prepared following a previously described methodology.^55^ Briefly, dry lipid films were obtained by mixing DOPC, DOPE, and DOPS stocks in chloroform (10 mg/mL) at a ratio of 5:3:2 (w/w) under an argon atmosphere. Chloroform was then evaporated under an N_2_ flow and 2 h vacuum. The dry films were stored under argon at −20 °C. Multilamellar vesicles were prepared at the desired final concentration by hydrating the dry films in UW using eight cycles of 60 s in an ultrasonication bath and 30 s of vortexing at 22 °C. Four 5 min cycles of tip probe sonication (UP50H, Hielscher Ultrasonics GmbH, Germany) (including 2 min in between to cool down the sample and minimize lipid damage by heat^56^) were used to obtain SUVs with final sizes of around 30 nm. The liposome charge and size were characterized by DLS following the same protocol as for NP characterization.

### QCM-D

QCM-D measurements were performed using a QSense analyzer (Biolin Scientific, Sweden), and silica-coated sensors (QSense QSX 303 SiO_2_, 4.95 ± 0.05 MHz, 14 mm diameter, 0.3 mm thickness, and a mass sensitivity factor of 17.7 ng/cm^2^) (Figure S6) were used as substrates for DOPC/DOPE/DOPS (5:3:2) bilayers as described previously.^57^ Cleaning and the rest of the experimental procedure were performed as described previously.^55^ Briefly, the tubing and the cells were thoroughly cleaned with a 2% Hellmanex solution and multiple UW rinses, combined with bath sonication, followed by rinsing with pure ethanol and drying under an N_2_ flow. Before the cell assembly, the silica surfaces were washed in 2% Hellmanex, UW, and finally ethanol, dried with N_2_, and plasma-cleaned (Model PDC-32G, Harrick Plasma, USA) for 2 min.

During the experiments, a flow rate of 0.1 mL/min (Ismatec IPC 4-channel peristaltic pump, Cole-Parmer GmbH, Germany) and a constant temperature of 25 °C were set. To form the supported lipid bilayers (SLBs), first UW was pumped until reaching a stable baseline for at least 5 min. To promote bilayer formation in the presence of anionic lipid headgroups, prior to injecting the SUV suspensions, the temperature was increased to 37 °C and a solution of 2 mM CaCl_2_ was flushed for 5 min. This was a necessary step to obtain stable bilayers made of negatively charged lipids and has previously been demonstrated to result in full surface coverage and largely defect-free bilayers in related systems.^58,59^ Efficient bilayer formation, in turn, is important for reducing any direct influence of the negatively charged silica surface on NP interactions, as reported for positively charged peptides.^60^ After bilayer formation, excess liposomes and CaCl_2_ were removed by extensive rinsing with UW, after which the temperature was decreased to 25 °C. SUV deposition, rupture, and full bilayer formation were confirmed by frequency changes (Δ*F*) of −23 ± 1 Hz and dissipation changes (Δ*D*) of (0.25 ± 0.25) × 10^–6^ with respect to UW (Figure S2). Following bilayer formation, 150 mM NaCl buffered with 5 mM HEPES at pH 7.4 was pumped for 10 min, and the NP sample was pumped for 1 h onto the SLBs at 100 ppm in the same saline buffer. For quantification, the seventh harmonic was chosen for maximum data robustness.^24,55^ When the adsorbed PS NPs formed a homogeneous film and the dissipative energy losses were small (Δ*D*/Δ*F* < 1 × 10^–7^ Hz^–1^ for all overtones,^61^ normalized Δ*F* equal for each overtone^62^), the Sauerbrey equation was used to calculate the deposited mass Δm from the frequency variations:^63−65^
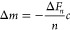
where Δ*F_n_* is the measured frequency shift at overtone number *n* and *C* is the mass sensitivity constant that equals 17.7 ng/Hz cm^2^. Measurements were conducted in triplicate and reported as the mean ± standard deviation.

### Planar Bilayer Formation and Electrical Measurements

Planar membranes were obtained by apposition of two lipid monolayers in a Teflon chamber separated in two compartments of 1.6 mL each (so-called cis and trans) by a 15 μm-thick Teflon film with a central orifice of 70–100 μm diameter, using a solvent-free modified Montal–Mueller technique^66,67^ (Figure S7). In brief, the lipid was prepared by dissolving DOPC/DOPE/DOPS (5:3:2 w/w) in pentane at 5 mg/mL after chloroform evaporation under argon. The orifice was pretreated with a 1% solution of hexadecane in pentane. After pentane evaporation, both the cis and trans compartments were filled with the desired saline buffer using syringes, and 10–20 μL aliquots of the lipid solution were deposited on the surfaces of both subphases to form two monolayers. The solvent was allowed to evaporate for 10 min. Then, the level of the solution in each compartment was raised above the hole, so the planar bilayer was formed by monolayer apposition at both sides of the hole. PS NP stock solutions prepared at 10,000 ppm were sonicated and vortexed to ensure full dispersion before their addition to the cis side of the chamber, and they were added at a final concentration of 100 ppm.

To record NP-induced electrical currents through the planar membrane, once the particles were added, a periodic voltage sweep was applied, composed of 70 mV for 24 s followed by −70 mV for another 24 s, as described in ref (25). This voltage difference was applied using home-built Ag/AgCl electrodes in 2 M KCl with 1.5% agarose bridges assembled within standard 250 μL pipette tips. The potential was defined as positive when it was higher at the side of NP addition (cis side), whereas the trans side was set to ground. An Axopatch 200B amplifier (Molecular Devices, Sunnyvale, CA) in the voltage clamp mode was used to measure the current and the applied potential. Current was filtered with a 5 kHz 8-pole in-line Bessel filter and digitized with a Digidata 1440A (Molecular Devices, Sunnyvale, CA) at a 20 kHz sampling frequency. The membrane chamber and the headstage were isolated from external noise sources with a double metal screen (Amuneal Manufacturing Corp., Philadelphia, PA). Conductance values were obtained from current measurements in symmetrical salt solutions of 150 mM NaCl buffered with 5 mM HEPES at pH 7.4 with NPs added at a final concentration of 100 ppm. The conductance values were evaluated using the Gaussian fit tool of Clampfit 10.7 (Molecular Devices, Sunnyvale, CA). The cation versus anion preference of NP-induced currents was assessed by measuring the reversal potential (RP), that is, the applied voltage needed to precisely cancel the current measured when a given salt concentration gradient is applied to the system. For these RP experiments, planar membranes of either DOPC/DOPE/DOPS (5:3:2 w/w) or DOPC/DOPE (7:3 w/w) were formed under fivefold (50 mM/250 mM NaCl) concentration gradients, and the net ionic current obtained was manually set to zero by adjusting the applied potential. This potential was then corrected by subtracting or adding (depending on the salt gradient orientation) the liquid junction potential of the electrode salt bridges^68^ to obtain the RP. The measured RP was converted into a channel permeability ratio *P*_+_/*P*_–_ by means of the Goldman–Hodgkin–Katz equation.^69^ In these assays, the final NP concentration at the cis side was increased to 200 ppm for PN-NH_2_ and to 500 ppm in the case of PS and PS-COOH to ensure enough pore formation events.

## Results and Discussion

### NP Aggregation, Size, and ζ-Potential

**Figure 1:**
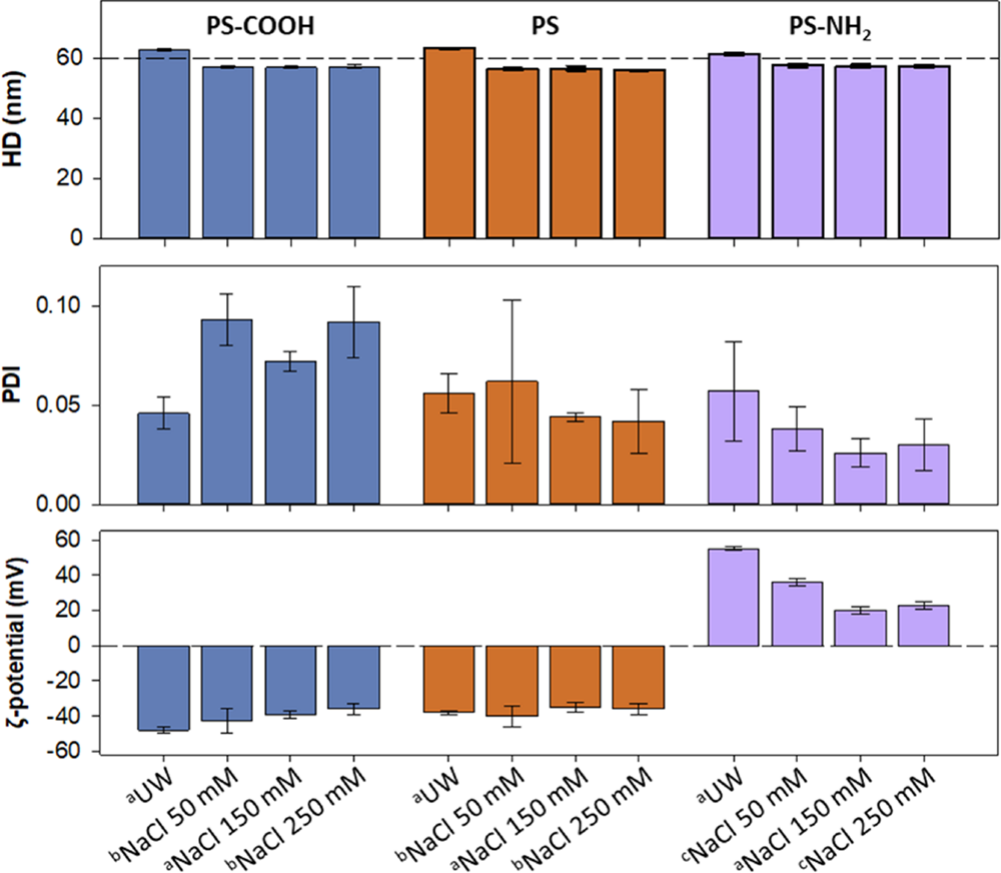
DLS characterization of PS NPs at different concentrations in UW and in NaCl buffered with 5 mM HEPES at pH 7.4, showing the hydrodynamic diameter (HD, upper panel), polydispersity index (PDI, middle panel), and ζ-potential (lower panel). ^*a*^100 ppm; ^*b*^500 ppm; and ^*c*^200 ppm.

We first used the DLS to characterize the colloidal properties of NPs under different conditions, as shown in Figure 1. Results in UW confirm the manufacturer data, and the three NP suspensions shown are essentially monodispersed (PDI < 0.2) with an average diameter of approximately 60 nm. In NaCl solutions at pH 7.4, the NPs showed a slight reduction (∼10%) of their size while monodispersity was maintained. Importantly, no aggregation was found for any NP under the conditions investigated during the time period (1–2 h) comparable to that of QCM-D and electrophysiological experiments reported later in the study. To highlight the importance of this initial characterization, note that NPs with amidine functionalization were discarded from our study because DLS measurements showed aggregation in NaCl solutions (Figure S1).

As regard to ζ-potential, PS-COOH showed negative values whereas PS-NH_2_ displayed positive ones, as expected from the presence of either negatively charged carboxyl groups (PS-COOH) or positive amines (PS-NH_2_). However, plain (according to manufacturer’s nomenclature) PS NPs yielded negative zeta potential values similar to those found for PS-COOH, which is attributable to the sulfate groups resulting from the persulfate initiator used in the PS emulsion polymerization process as reported by the manufacturer (Magsphere Inc.).^70^

### NP Interactions with Supported Lipid Membranes

To assess the interaction between PS NPs and model lipid membranes, we employed QCM-D, a technique that allows real-time monitoring of the formation and stability of thin films.^24,28,57,61,63,65,71−73^ We formed DOPC/DOPE/DOPS (5:3:2 w/w) SLBs based on the phospholipid headgroups mainly composing cell membranes such as those of the endoplasmic reticulum, mitochondria, and lysosome.^33−35^ Of note, this composition is similar to the membrane of the *Artemia franciscana* brine shrimp eyes,^36−38^ a species widely used in toxicology and particularly to assess the toxicity of plastic NPs.^15,39,74−76^

**Figure 2:**
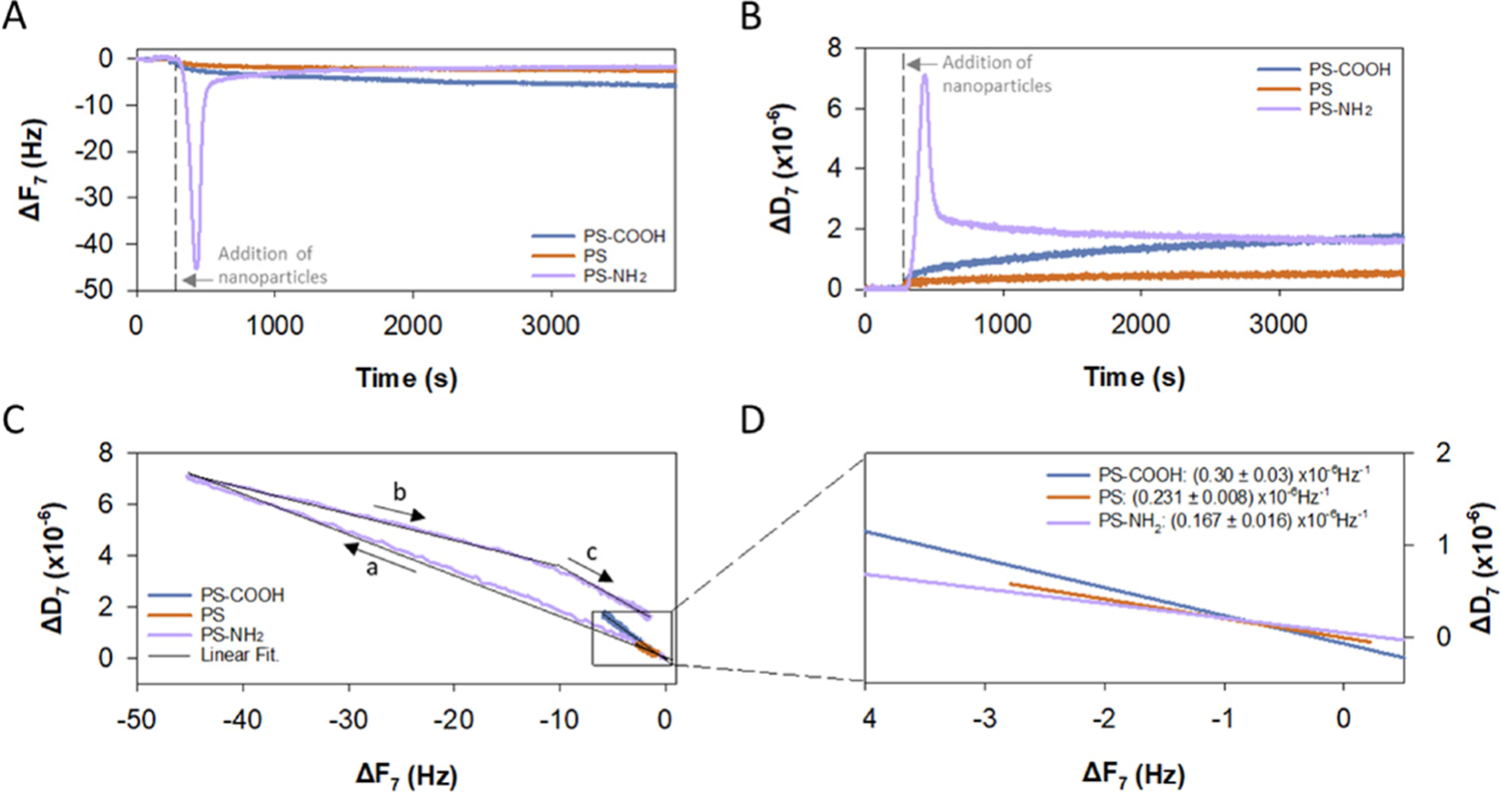
Representative QCM-D measurements of changes in frequency (Δ*F*) (A) and dissipation (Δ*D*) (B) at the seventh overtone during deposition of a NP suspension (in 150 mM NaCl with 5 mM HEPES at pH 7.4) on 5:3:2 DOPC/DOPE/DOPS bilayers. A plot of Δ*D* vs Δ*F* (C) and comparison of the obtained slopes in panel (C) (absolute values; slope *a* was considered for PS-NH_2_) (D). In panels (A and B), the reference line indicates the start of NP suspension addition (*t* = 300 s). In panel (C), the arrows indicate the evolution of slopes with time.

**Figure 3:**
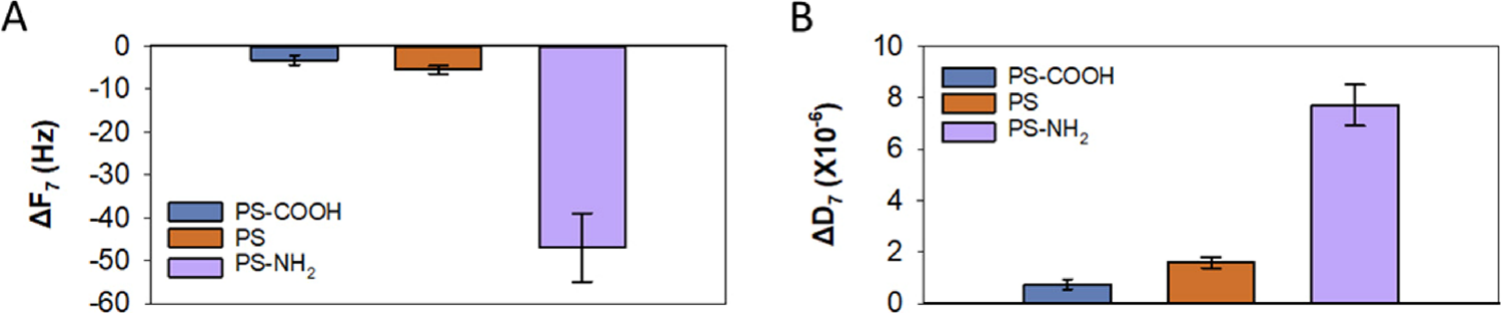
QCM-D maximum change in frequency and dissipation measured for the seventh overtone after PS NP deposition in 150 mM NaCl with 5 mM HEPES at pH 7.4 on a supported 5:3:2 DOPC/DOPE/DOPS bilayer. The maximum change of PS-COOH and PS corresponds to the final equilibrium value (*n* = 3).

After SLB formation at 37 °C, full surface coverage and absence of noncollapsed SUVs were confirmed (Figure S2) in line with previous results^57,77,78^ and studies where neutron reflectometry was used to demonstrate defect-free bilayers.^55,79^ The addition of different PS NPs to such preformed lipid bilayers (performed at 25 °C) induced an increase in mass of the resonator detectable by a decrease in frequency (Δ*F*) and an increase in dissipation (Δ*D*) (Figures 2A,B and 3). This indicates that all studied PS NPs are able to reach the bilayer surface and interact with the SLB, with the positively charged PS-NH_2_ showing the largest changes (by an order of magnitude) both in frequency and dissipation (Figure 3).

Time evolution of Δ*F* and Δ*D* shows additional qualitative trends not visible in Figure 3. PS and PS-COOH (Figure 2A,B) show small changes (a decrease in frequency and an increase in dissipation) that increase gradually with time until reaching a quasi-equilibrium (Figure S3). In contrast, PS-NH_2_ shows nonmonotonical dramatic changes in both frequency and dissipation. Thus, a quick initial peak is followed by a slow decay, leading to saturation values of Δ*F* = −1.2 ± 0.7 Hz and Δ*D* = (1.3 ± 0.3) × 10^–6^, comparable to those of the other two NPs (Figures 2A,B and 3).

Such contrasting features are visible in Δ*D* versus Δ*F* plots (Figure 2C) that may reflect the viscoelastic properties of the adsorbed layers and also hydrodynamic effects.^80^ PS and PS-COOH NPs display one single Δ*D*/Δ*F* slope, whereas PS-NH_2_ NP presents a more complex pattern with different regions (see Figure 2C). Large values of Δ*D*/Δ*F* ratios are commonly associated with soft, flexible, and hydrated film conformations, whereas low values are usually observed in dehydrated rigid structures.^62,71,81−83^Figure 2D shows a zoomed-in section of Figure 2C for the lowest values of Δ*D* and Δ*F*. Considering that the Δ*D*/Δ*F* slope in Figure 2D is lower for PS-NH_2_, followed by PS and PS-COOH, and the fact that the film stiffness increases with the strength of NP adsorption, we conclude that PS-NH_2_ has the highest affinity for the bilayer followed by PS and PS-COOH.

The complex behavior of PS-NH_2_ deserves further explanation. A close inspection of Figure 2C (arrows showing the time evolution) indicates the occurrence of at least two regimes. The first of these (Figure 2C arrow a) suggests the continuous incorporation of positive NPs to the lipid film causing destabilization of the initially confluent lipid bilayer by interfacial reactions such as osmotic dehydration.^81,84^ The second regime is characterized by gradients of Δ*D*/Δ*F* having an opposite sign to the first stage with two different slopes (Figure 2C arrow b and arrow c) suggesting a transition to a more dissipative structure than in the other NPs that results from the instability of the rigid bilayer created after the initial stage (see Figure S4 for additional details). Because PS-NH_2_ displays a much higher dissipation than that seen for PS and PS-COOH, we believe that the observed changes are not purely hydrodynamic effects but instead attributable to a different interaction with the lipid bilayer. Thus, we hypothesize that PS-NH_2_ particularly enhances lipid extraction from the supported film^27^ and/or the creation of water defects that induce lipid reorganizations^32,85^ (Figure S5). Such extraction of anionic phospholipids and lipid removal have been observed also for other cationic NPs.^27,86,87^ Overall, the finding that the final Δ*D* after NP exposure was higher than the initial value indicates that soft films of an inhomogeneous structure are obtained for the three PS NPs under study (Figure 2C,D). Further analysis of the interaction by complementary techniques such as scattering-based microscopy methods,^88^ which may provide additional information on NP adsorption into the SLBs, are out of the scope of the present study.

### NP-Induced Currents in Planar Model Membranes

To evaluate potential membrane permeabilization, we next prepared planar bilayers by apposition of two monolayers made of DOPC/DOPE/DOPS in a 5:3:2 ratio (w/w) in 5 mM HEPES at pH 7.4 with 150 mM NaCl following the Montal–Mueller technique.^67^ This approach provides very stable solvent-free membranes in which spontaneous permeabilization in the absence of NPs was found to be nonexistent. After NP addition to the cis chamber of the cell, voltage differences of ±70 mV were applied between both sides of the cell in periodic intervals and the transmembrane current was measured. By using this protocol, we investigate the influence of voltage polarity and NP charge on the NP–membrane interaction.^25^ Also, we simulate, as a first approximation, the conditions of living cells where the concerted interplay of ion channels generates dynamic changes in both membrane potential (∼ ±100 mV)^89,90^ and ionic fluxes.^91,92^

**Figure 4:**
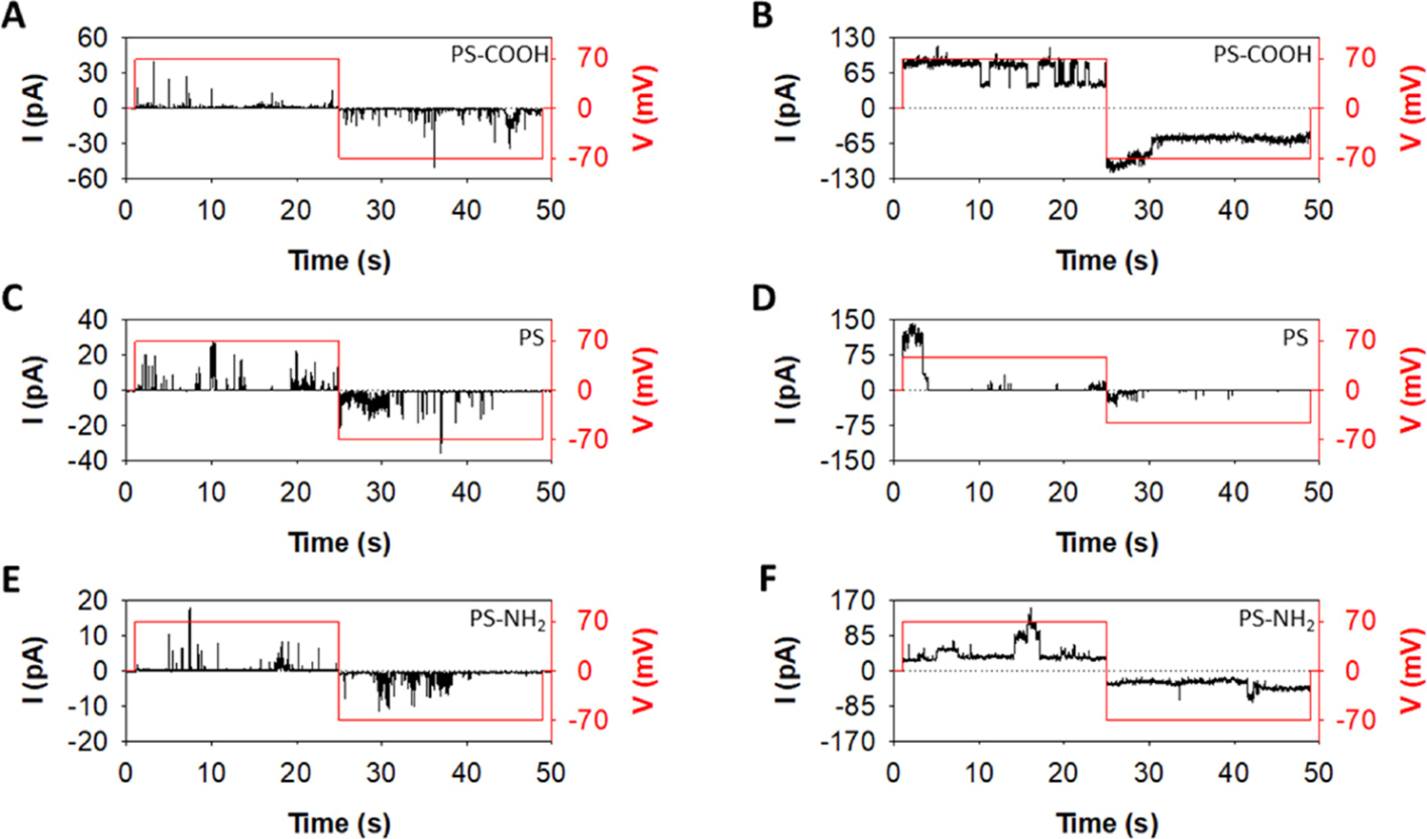
Representative currents induced by PS-COOH (A,B), PS (C,D), and PS-NH2 (E,F). Panels (A,C,E) show how addition of NPs induces transient current spikes so short-lasting that quantification of the average current is not possible. Panels (B,D,F) display example traces in which NP addition yields stepwise current jumps of variable but well-defined conductance levels.

Figure 4 shows the representative traces of the ion currents induced by PS-COOH (A,B), PS (C,D), and PS-NH_2_ (E,F) displaying a variety of current levels that fluctuate with time. For each PS NP, either transient current spikes (Figure 4A,C,E) or well-defined stepwise current jumps (Figure 4B,D,F) were found for both voltage polarities, similar to those caused by proteins forming membrane defects such as aqueous pores.^21^ Importantly, all NPs considered in our study are able to induce membrane permeability without leading to global membrane disintegration. The finding of the conservation of membrane integrity, a capital issue to distinguish between NP binding and internalization in cells,^23^ is in line with the previous findings for the interaction between PS particles of different sizes (from 20 nm to ∼2 μm) and giant unilamellar vesicles.^93−95^

In a previous study following a similar voltage protocol as the one used here,^25^ PS-NH_2_ induced progressively increasing currents that finally lead to membrane disintegration, whereas PS-COOH failed to induce membrane permeability.^25^ Such differences with our present findings could originate either from differences in bilayer preparation (liposome addition to parallel array platforms there) or from differences in membrane compositions (the previous study including also cholesterol and cerebroside^25^).

### Assessment of Membrane Permeabilization by NPs

**Figure 5:**
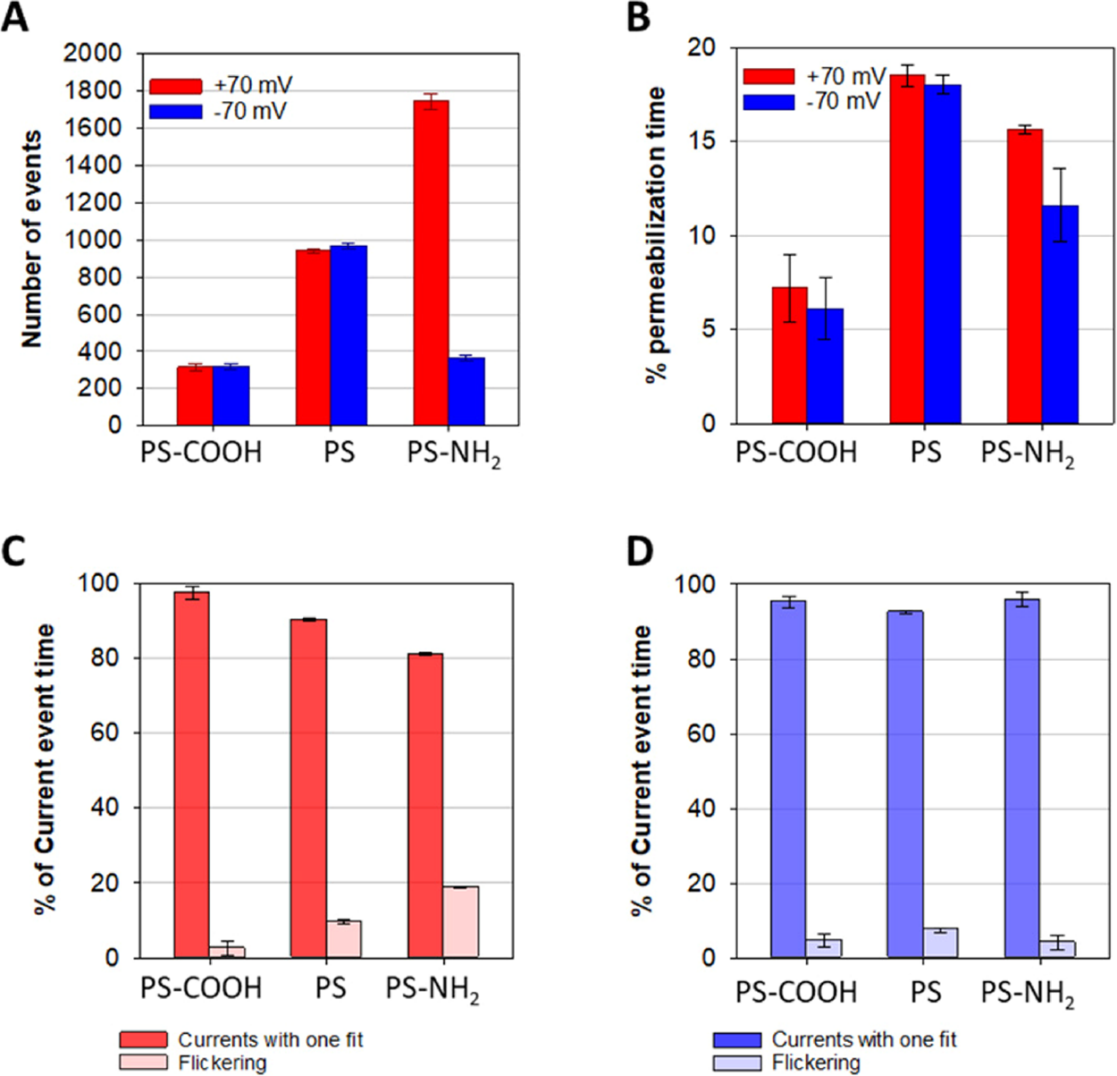
Number of events (A) and percentage of recorded time with nonzero current levels (% permeabilization time, B). Percentage of current time with defined conducting levels and flickering events at positive (C) and negative (D) applied voltages. Errors were calculated using error propagation.

Given the similarity between the NP-induced current traces and those caused by membrane proteins, we use quantification methods employed in the analysis of ion channel activity, such as the number of events. An event is the time period of a current trace with a well-defined conducting level (i.e., the histogram of current values can be represented by a single peak) (see Experimental Section). The number of current events induced by each type of NP is shown in Figure 5A to clearly illustrate the results. Thus, PS-COOH NPs show little membrane disruption, without any observable influence of voltage polarity. PS-NH_2_ under *V* < 0, in turn, shows a number of events compared to PS-COOH. In contrast, for positive voltages, in which the positively charged NPs are forced to go toward the bilayer, the number of events displays a fivefold increase compared to that observed for the opposite polarity. Nonfunctionalized PS, finally, represents an intermediate case, showing no dependence on the sign of the applied voltage.

The time variability of current traces in Figure 4 suggests that the number of events shown in Figure 5A could be insufficient to assess the membrane permeability. That is, one type of NP displaying many events of short duration may have a similar permeabilization capacity to another type of NP with few, but longer, events. To address this issue, the fraction of the total experimental time in which NP-induced currents were present is shown in Figure 5B. PS-COOH NPs show the lowest values (∼6%) whereas nonfunctionalized PS NPs show the largest ones (almost 20%), being largely insensitive to the voltage polarity, in line with the data in Figure 5A.

The case of PS-NH_2_ is particularly complex as the large number of events at the positive voltage in Figure 5A does not lead to longer permeabilization times in Figure 5B. Indeed, PS-NH_2_ NPs in Figure 5B show lower time fractions than PS, and the dependence on voltage polarity is clearly reduced in comparison with Figure 5A. To clarify this point, we next classified the events making a distinction between those with well-defined duration and the so-called flickering^96^ for positive and negative applied voltages (Figure 5C,D). In practice, flickering corresponds to fast transitions that are unresolved because their life-times are comparable to the sampling time of our protocol (0.05 ms)^97^ (see Experimental Section). Interestingly, PS-NH_2_ NPs at *V* > 0 show a larger occurrence of flickering, explaining why a large number of events (1744 ± 44, Figure 5A) does not translate to a large time with current events (15.6 ± 0.23%, Figure 5B).

**Figure 6:**
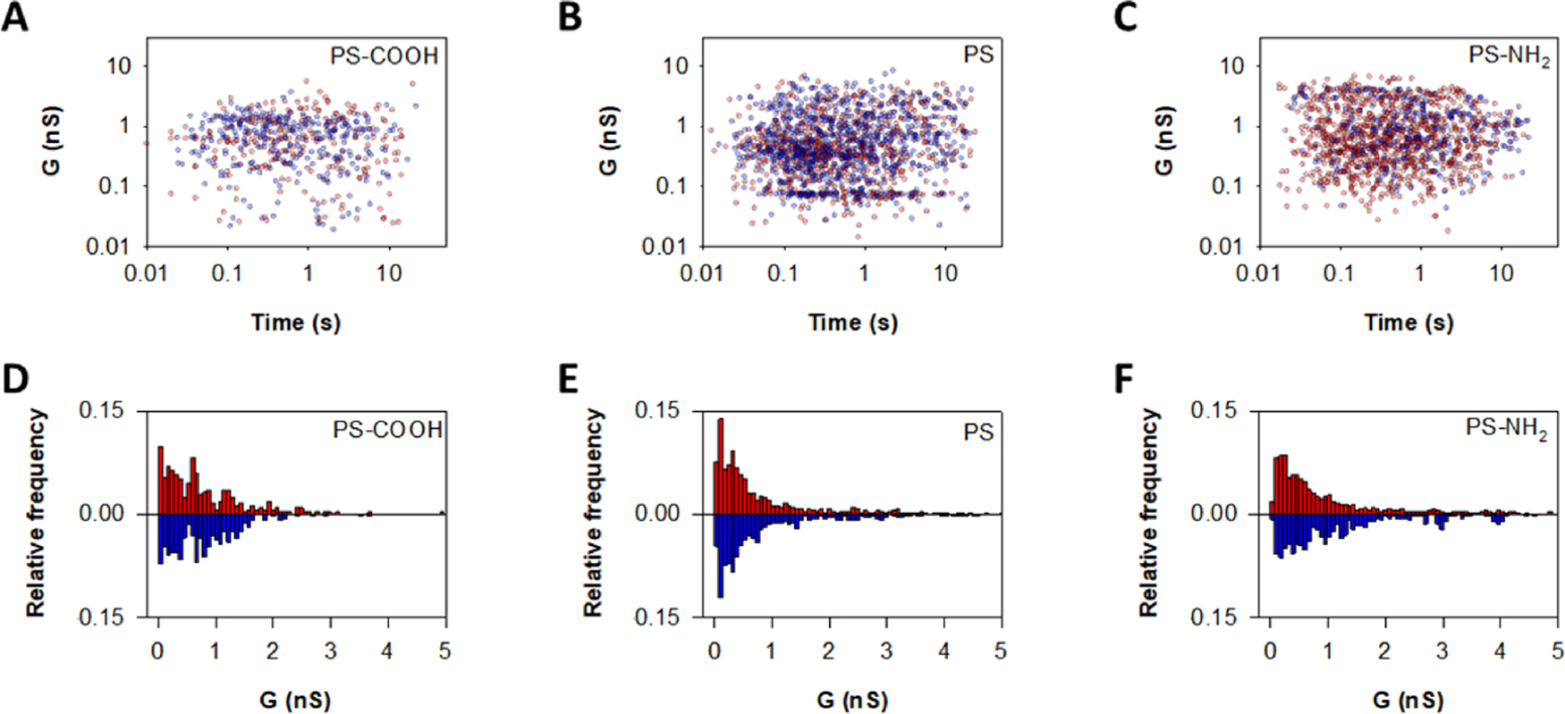
Correlation between event conductance and time length for PS-COOH (A), PS (B), and PS-NH_2_ (C). NP-induced conductance levels at positive (red) and negative (blue) applied voltages for PS-COOH (D), PS (E), and PS-NH_2_ (F).

Figure 6A–C shows that there is no correlation between the measured conductance level (*G*) and the corresponding lifetime, regardless of the NP type and voltage polarity. The randomness of *G* versus *t* distributions emphasizes that the observed transmembrane currents correspond to stochastic transitions (successive openings and closings) and not to progressive membrane disintegration. Histograms of conductance values in Figure 6D–F for NP PS-COOH, PS, and PS-NH_2_ show considerable dispersion, with the most probable conductance peak at around *G* ∼ 20–50 pS and secondary peaks at higher conductance values that extend to several nS. As the first approximation, the pore conductance can be written as *G* ∼ κπr^2^/L where κ is the electrolyte conductivity (κ ∼ 1.7 S/m for 150 mM NaCl and KCl at pH 7.4) and *L* ∼ 5 nm is the pore length assuming that it spans across the distance of the lipid bilayer.^98^ This allows a rough estimation of the characteristic pore radius (*r*), corresponding to *r* ∼ 0.3 nm for *G* ∼ 50 pS. The upper limit of our conductance measurements (*G* ∼ 1 nS) would correspond to *r* ≥ 1 nm. Such scattered values in pore dimensions are characteristic of systems forming disordered channel structures where there is not a unique pore configuration but rather a variety of arrangements, as seen for viral proteins^43−51^ cell-penetrating peptides,^99^ and other small charged peptides.^42,52,100^ Note that the myriad of pore configurations, as opposed to that of well-defined canonical ion channels,^96^ is seen in a dual way: as a static disorder (existence of different conductive levels in the traces) but also as a dynamic disorder (variation of current levels with time, shown in Figure 6A–C).^101^

**Figure 7:**
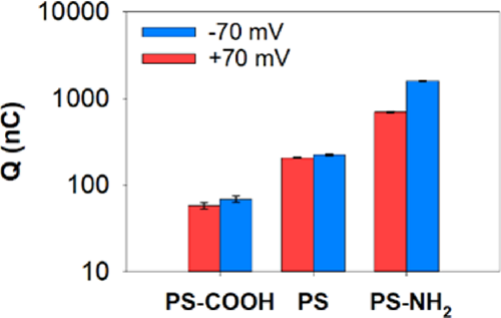
Transferred charge at positive and negative applied voltages. *Y*-axis is shown in a logarithmic scale. Errors were calculated using error propagation.

A combined measurement of the membrane-disrupting activity of PS NPs could come from the transferred charge *Q* calculated as the integral of the current over time as shown in Figure 7. As seen from the image, the transferred charge for PS is almost four times higher than that for PS-COOH, as could be expected because PS shows a higher number of events and longer times of current events than PS-COOH. Also, both PS and PS-COOH display only minor differences with the voltage polarity in transferred *Q*. PS-NH_2_ is again different because of the larger number of events for *V* > 0 with respect to *V* < 0 (1774 vs 366), which, however, does not imply a higher transference of charge. In fact, it is just the other way around (696 vs 1587 nC). This was motivated by the larger occurrence of flickering at *V* > 0 (Figure 5C,D).

The combined findings of Figures 5–7 reveal that PS-COOH NPs are the least membrane-disruptive of the three NPs investigated, yielding the lowest values in number of events, permeabilization times, and transferred charge. Also, we observe that PS has much higher affinity for DOPC/DOPE/DOPS (5:3:2) membranes than PS-COOH. Considering that both displayed very similar ζ-potentials in Figure 1 and hence similar negative charges, the differences between PS and PS-COOH may have their origin in NP hydrophobicity.^18,24,102,103^ In line with this, in our hydrophobicity assay (see Supplementary Information), the surface hydrophobicity was found to be higher for PS than for PS-COOH (Table S1).

Another important finding is that PS-NH_2_ NPs display the highest disruption capacity if all factors (Figures 5–7) are taken together. Thus, PS-NH_2_ NPs have a similar permeabilization time to PS, but the transferred charge *Q* is much higher, especially in the voltage polarity where PS-NH_2_ NPs are not driven toward the membrane but in the opposite direction. Our results suggest that other factors than purely electrostatic interactions between positive PS-NH_2_ and negatively charged lipids^25^ play a major role. As shown in Table S1, the surface hydrophobicity of PS-NH_2_ is comparable to that of PS and much larger than that for PS-COOH. Interestingly, Mielke and Zimchl^102^ reported that the particle–solvent behavior at the interface of hydrophobic materials such as PS NPs is regulated by the hydrophobic/hydrophilic balance that can be maintained (PS-NH_2_) or altered (PS-COOH) in the functionalization process.^104^ Finally, note that there is a qualitative agreement between QCM-D and electrophysiology about the higher membrane-disruptive capacity of PS-NH_2_. However, both techniques are not directly quantitatively comparable because QCM-D encompasses horizontal SLBs formed on glass with NP suspensions being pumped constantly, whereas electrophysiology involves free-standing vertical lipid bilayers and the addition of discrete aliquots of NPs.

### Ion Selectivity of NP-Induced Pores

One intriguing issue arising from previous sections is the structure of the permeation pathways created by ∼60 nm NPs on ∼5 nm-thick lipid bilayers. At this point, it is unclear whether NPs are structurally involved in the formation of membrane defects or pores^32^ or if their action resembles to the “detergent-like mechanisms” invoked for proteins that just disrupt the lipid packing creating hydrophilic pores.^105,106^

To elucidate how NPs and membrane lipids contribute to the pore structure, we next performed selectivity experiments using a fivefold concentration gradient of NaCl (50 and 250 mM) buffered with 5 mM HEPES at pH 7.4. As shown in Figure 1, there was no NP aggregation under these conditions. We performed experiments using two different membrane compositions, the physiologically relevant DOPC/DOPE/DOPS at a ratio of 5:3:2 (w/w) with 20% negative charge and a neutral membrane DOPC/DOPE at a ratio of 7:3 (w/w).

**Figure 8:**
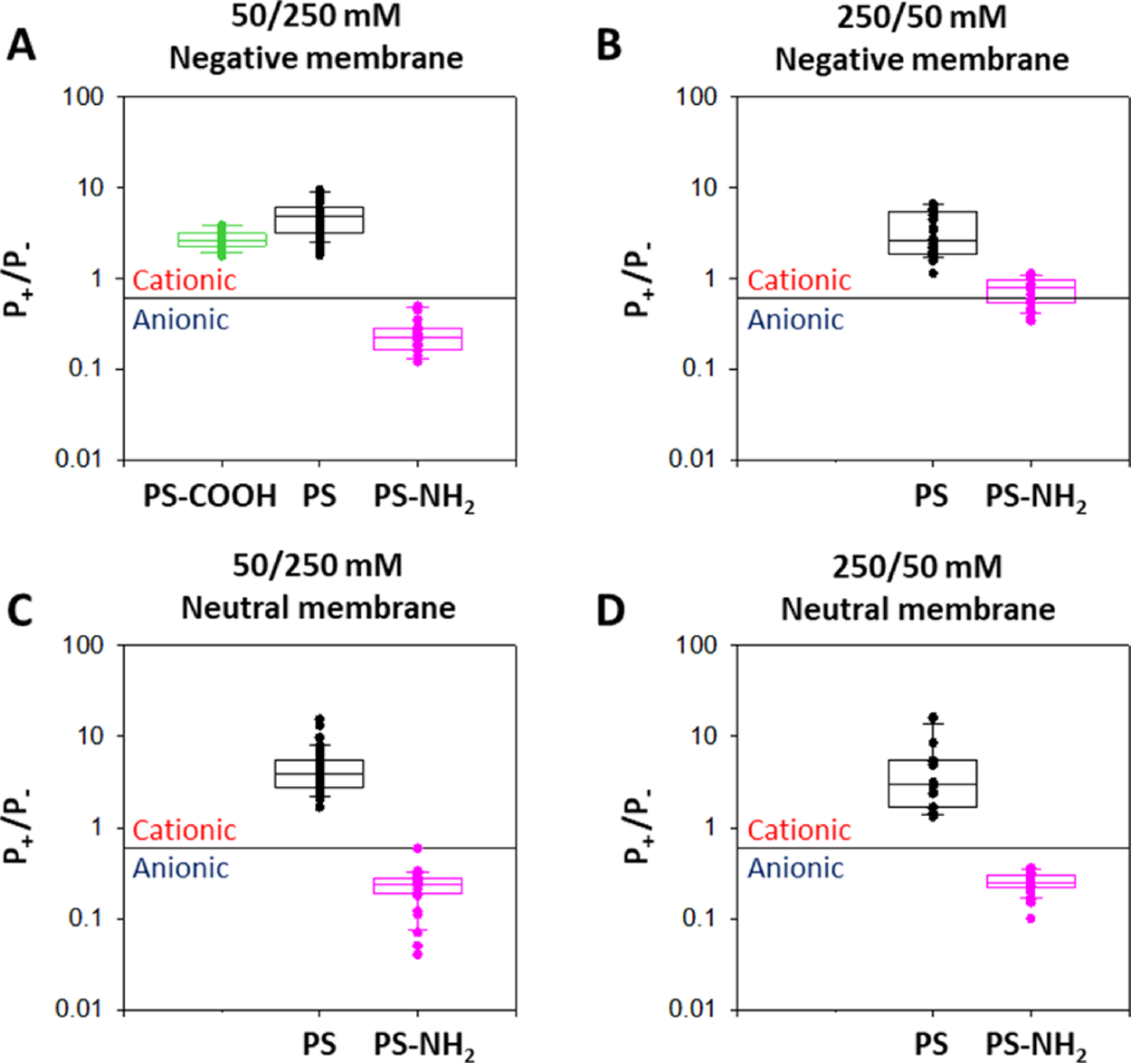
Ion selectivity of NP-induced pores under two salt gradients and two membrane lipid compositions. (A) 50/250 mM NaCl in DOPC/DOPE/DOPS (5:3:2). (B) 250/50 mM NaCl in DOPC/DOPE/DOPS (5:3:2). (C) 50/250 mM NaCl in DOPC/DOPE (7:3). (D) 250/50 mM NaCl in DOPC/DOPE (7:3). All salt solutions were buffered with 5 mM HEPES at pH 7.4. In all panels, the solid lines indicate the limit between cationic and anionic selectivities, located at a *P*_+_/*P*_–_ of 0.6 due to the different diffusivities of Na^+^ and Cl^–^ ions. Data are shown as box plots, where the boundary of the box closest to zero indicates the 25th percentile, a line within the box marks the median, and the boundary of the box farthest from zero indicates the 75th percentile. Solid circles correspond to individual experiments. Error bars indicate SD (*n* ≥ 20).

Figure 8A shows the measured selectivity (shown as the permeability ratio, *P*_+_/*P*_–_) when a charged membrane (DOPC/DOPE/DOPS at a ratio of 5:3:2 (w/w)) is placed under a NaCl concentration gradient of 50/250 mM and PS-COOH, PS, or PS-NH_2_ NPs are added to the diluted side. Under these conditions, both PS-COOH and PS NPs create pores with preference for cations, which is consistent with their negative ζ-potential (Figure 1) but also with the negative charge of the membrane. However, experiments with PS-NH_2_ NPs show a preference for anions, incompatible with the membrane charge but fully compatible with the positive net charge of the aminated NP revealed by the corresponding ζ-potential (Figure 1).

Figure 8B displays the selectivity obtained with experiments performed as in Figure 8A but with the opposite salt concentration gradient (250/50 mM) for NPs added to the concentrated side. Under these conditions, we did not obtain permeabilization events with PS-COOH despite increasing the NP concentrations five times up to 500 ppm. In contrast, PS NPs yielded pores with a cationic selectivity similar to that in the 50/250 mM salt gradient (Figure 8A). Importantly, experiments with PS-NH_2_ NPs display scattered values that range from anionic selectivity to cationic one (Figure 8B). Positively charged PS-NH_2_ NPs alone cannot account for a cation-selective pore, indicating that the negatively charged lipids present in the membrane must participate in the pore structure similar to membrane proteins that assemble with lipids to form joint proteolipidic structures.^42−52^

To inspect the role of lipid charge in the pore selectivity, we also performed experiments in neutral membranes (DOPC/DOPE 7:3 (w/w), Figure 8C,D). PS NPs induced cation-selective pores that were slightly less selective than those in the experiments with a charged membrane, suggesting that both the NP charge and the membrane charge contribute to the overall selectivity. In addition, PS-NH_2_ generated anion-selective currents under the two orientations of the concentration gradient explored (50/250 mM (Figure 8C) and 250/50 mM (Figure 8D)). The fact that we did not find any cation-selective pore induced by PS-NH_2_ for neutral membranes as in Figure 8B validates the hypothesis that the lipid charge may be responsible for the cation-selective events obtained in that case.

Overall, our results confirm that PS NPs participate in the pore structure to form some kind of a combined arrangement together with lipid molecules that still is able to maintain global membrane integrity under a concentration gradient. In the conditions of our study (100 ppm of NPs and 20% of charged lipids in the 5:3:2 membrane), the selectivity of the induced pores is mainly ruled by the NP charge, including a small but measurable contribution from the membrane charge revealed by the comparison with control experiments in neutral membranes.

## Conclusions

We investigate how different PS NPs induce permeabilization of membranes made of physiologically relevant phospholipid compositions by combining complementary experimental techniques. The combined results of DLS, QCM-D, and electrophysiology experiments indicate that surface functionalization determines the NP–membrane interaction by a combination of different factors. The highest affinity for the negative bilayer found for PS-NH_2_ in QCM-D accompanied by the greatest disruption capacity in terms of electrophysiological parameters (number of events, permeabilization time, and transferred charge) suggest an electrostatic NP–membrane mechanism favoring positively charged NPs over negative ones (PS and PS-COOH). However, some findings put into question a purely Coulombic scenario. For instance, the asymmetry found regarding voltage polarity (the highest charge transference in PS-NH_2_ appears when NPs are driven outward from the membrane) suggests that lipid restructuring together with NP adhesion to the membrane surface could give rise to highly inhomogeneous charge distributions with non-Ohmic transport features. Thus, adsorption of positive PS-NH_2_ NPs onto a negatively charged membrane could yield bipolar-type charge distributions resembling p–n semiconductor junctions with nonlinear conduction.^107,108^ On the other hand, the pronounced differences between PS and PS-COOH with almost identical negative charges also reveal the importance of NP hydrophobicity, as confirmed by absorption measurements of Rose Bengal and in line with the previous studies.^24,102,104,109,110^

The fact that PS NP-induced pores maintain membrane integrity, as opposed to the progressive membrane disintegration found with other bilayer preparations and membrane compositions, has stimulating implications for NP–membrane interactions and nanoplastic toxicology. Concerning membrane architecture and dynamics, it is striking to note the difference in size between NPs (∼60 nm in our case) and typical membrane proteins inducing pores that approximately match the bilayer length (∼5 nm). Although the actual mechanism is still unknown, the NP adhesion leading to partial or total NP wrapping by lipids could involve, in the case of PS NPs, the formation of dissolved styrene clusters.^111^ However, it is unclear if these findings obtained for small PS NPs (600 styrene monomers) can be extrapolated to much larger particles of ∼60 nm.

**Figure 9:**
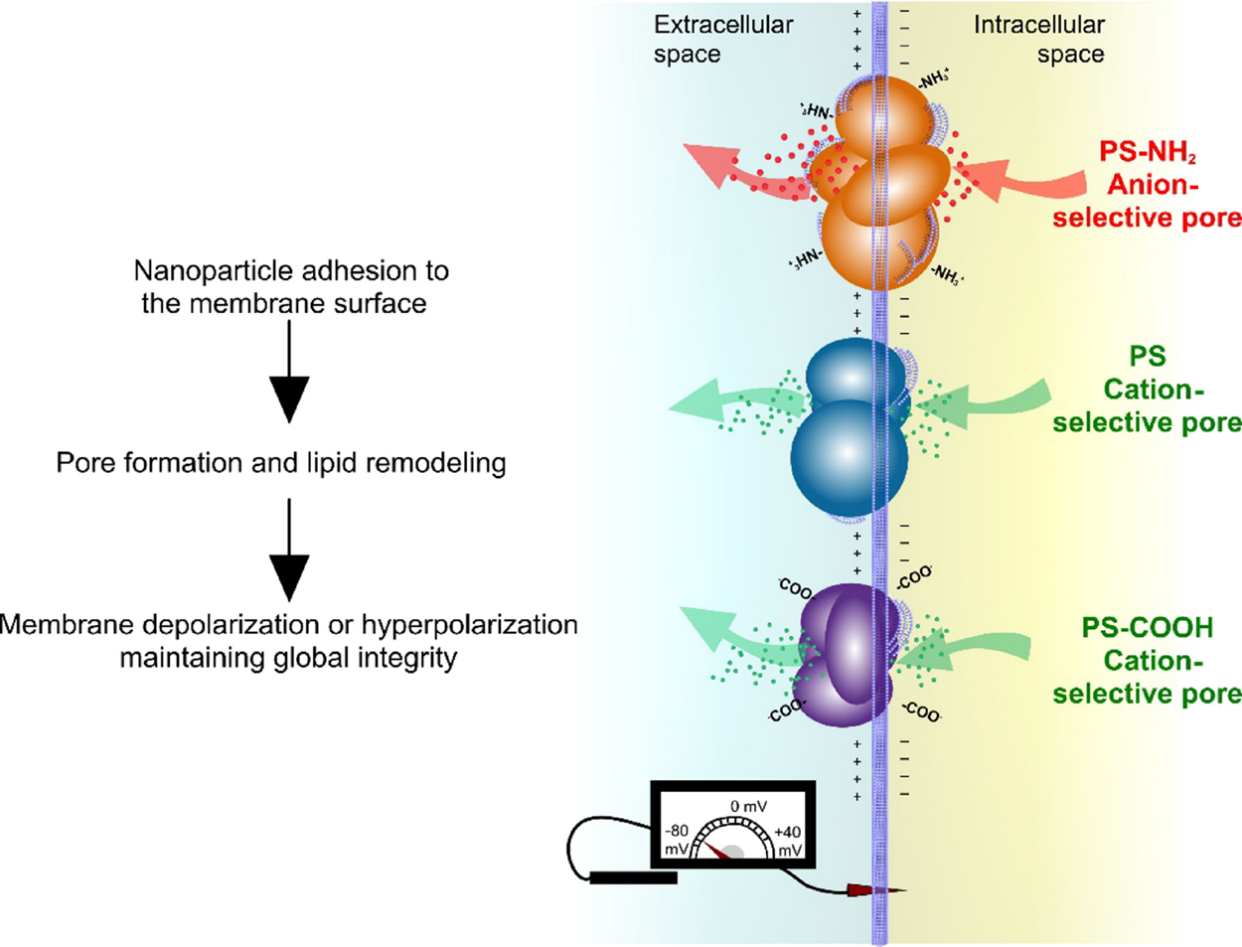
Cartoon illustrating the complex modulation of the cell transmembrane electric potential exerted by PS NPs.

As regard to NP-induced toxicity, the selective hydrophilic pores created by PS NPs point to a more complex modulation of transmembrane electric potential as summarized in the cartoon of Figure 9. Cell membranes are characterized by a net negative charge on the cytosolic side of the membrane (usually −40 to −70 mV^23^) that changes during cell proliferation and differentiation.^23,112^ Precedent toxicological studies have demonstrated that NPs could break the cell cycle by inducing not only the most common membrane depolarization^23,113^ but also hyperpolarization.^114,115^ The fact that cationic NPs are usually more toxic to cells than anionic ones^1,15,116,117^ could be tentatively explained in terms of our findings. We have shown that PS-NH_2_ has the most potent capacity of membrane disruption, pointing all our findings to membrane depolarization by different mechanisms. First, hypothetical internalization of positively charged PS-NH_2_ would decrease the negative membrane potential. Second, selective pores induced by PS-NH_2_ are cation-selective for the inward flow but anion-selective for the outward one, suggesting a highly asymmetric membrane charge distribution produced either from NP adhesion or from major lipid reorganizations. Note that both the entrance of positive charges and the exit of negative ones decrease the membrane potential. Interestingly, negatively charged PS and PS-COOH follow the opposite pattern: either by NP internalization or by the charge transferred by their induced selective pores, the membrane potential increases leading to hyperpolarization.

Overall, we hypothesize that PS NPs could act as artificial transmembrane ion transporters altering the cell homeostasis and ultimately inducing cell apoptosis^118,119^ by a combination of mechanisms that do not require membrane disintegration but more subtle mechanisms including the passive transport of charges and major membrane reorganizations by direct NP adhesion or NP-induced flip-flop.

## Supporting Information Available

DLS characterization of NPs, supported bilayer formation, NP deposition, hydrophobicity assays, QCM-D scheme, and planar bilayer formation (PDF)

## Abbreviations

PS NPs: polystyrene nanoparticles
DLS: dynamic light scattering
QCM-D: quartz crystal microbalance with dissipation monitoring
PS: plain polystyrene
PS-NH_2_: aminated polystyrene
PS-COOH: carboxylated polystyrene
DOPC: 1,2-dioleoyl-*sn*-glycero-3-phosphocholine
DOPS: 1,2-dioleoyl-*sn*-glycero-3-phospho-l-serine
DOPE: 1,2-dioleoyl-*sn*-glycero-3-phosphoethanolamine
HEPES: 4-(2-hydroxyethyl)-1-piperazineethanesulfonic acid
UW: ultrapure water
SUVs: small unilamellar vesicles
Δ*F*: frequency changes
Δ*D*: dissipation changes
SLB: supported lipid bilayers
PDI: polydispersity index
RP: reversal potential
*P*_+_/*P*_–_: channel permeability
GHK: Goldman–Hodgkin–Katz eq
*Q*: transferred charge
*G*: conductance
