# Supplementary material for: Surface-Functionalized Polystyrene Nanoparticles Alter the Transmembrane Potential via Ion-Selective Pores Maintaining Global Bilayer Integrity
Source: Langmuir. 2022 Nov 23;38(48):14837–49. doi: 10.1021/acs.langmuir.2c02487 (PMC9974068; doi:10.1021/acs.langmuir.2c02487)
Supplement: Supplementary file 1 — la2c02487_si_001.pdf [file la2c02487_si_001.pdf]

Supporting information

Surface-functionalized polystyrene nanoparticles alter transmembrane potential via pore formation maintaining global bilayer integrity

D. Aurora Perini[‡], Elisa Parra-Ortiz[†], Inmaculada Varó[§], María Queralt-Martín[‡], Martin Malmsten, [†,‹‹] and Antonio Alcaraz*[‡]

[‡]Laboratory of Molecular Biophysics, Department of Physics, Universitat Jaume I, 12071 Castellón, Spain

[†]Department of Pharmacy. University of Copenhagen, DK-2100 Copenhagen, Denmark

[§]Institute of Aquaculture Torre de la Sal (IATS-CSIC), 12595 Ribera de Cabanes, Castellón, Spain

[‹‹]Department of Physical Chemistry 1, University of Lund, SE-22100 Lund, Sweden

*Author to whom correspondence should be addressed. Electronic mail alcaraza@uji.es

TABLE OF CONTENTS

1. DLS Characterization of amidine nanoparticles size S2
2. Supported bilayer formation S3
3. Time derivate frequencies of nanoparticles deposition S5
4. Normalized frequencies and dissipation changes S6
5. Illustration of nanoparticles deposition S8
6. Hydrophobicity assay with Rose Bengal S9
7. Nanoparticles hydrophobicity results S10
8. QCM-D scheme S12
9. Planar bilayer formation S13

1. **DLS Characterization of amidine nanoparticles size**

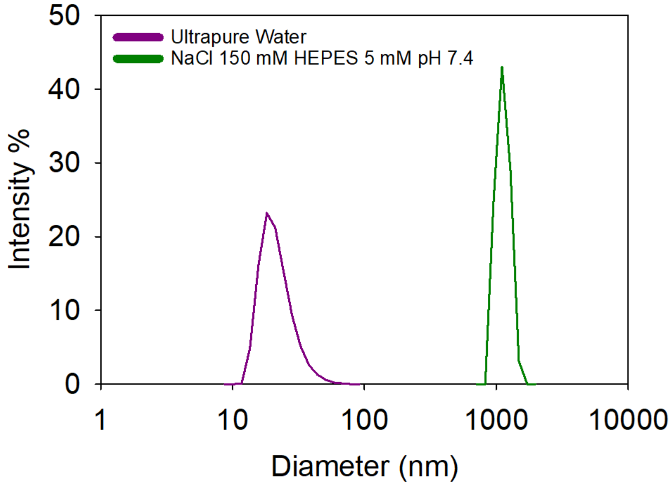

Figure S1. DLS size characterization of amidine nanoparticles at 100 ppm in Ultrapure Water (UW) and in NaCl 150 mM HEPES 5 mM pH 7.4.

2. Supported bilayer formation

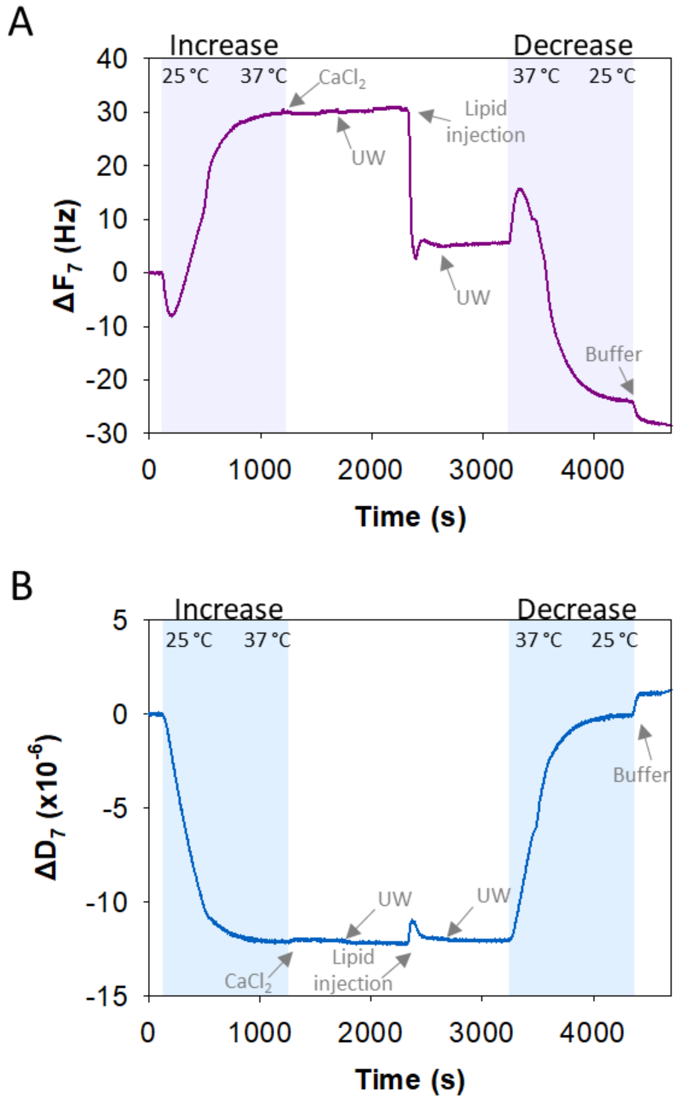

Figure S2. SUV deposition and resulting supported lipid bilayer formation shown as the 7th overtone of frequency (**A**) and dissipation (**B**) changes. A constant flow of rate of 0.1 mL/min was set. First, UW was pumped until reaching a stable baseline for at least 5 minutes. Second, the temperature was increased from 25 to 37 °C. Third, a solution of 2 mM $CaCl_2$ was flushed during 5 minutes. Fourth, UW rinsing to removed excess of $CaCl_2$. Fifth, injection the SUVs suspensions. Sixth, UW rinsing to removed excess of SUVs. Seventh, decrease the temperature from 37 to 25 °C. Finally, rising with buffer prior no nanoparticles addition. SUV deposition, rupture, and full

bilayer formation was confirmed by frequency changes (ΔF) of -23 ± 1 Hz and dissipation changes (ΔD) of $(0.25 \pm 0.25) \times 10^{-6}$ with respect to UW. (UW indicate ultrapure water; Buffer indicate NaCl 150 mM HEPES 5mM pH 7.4).

3. Time derivate frequencies of nanoparticles deposition

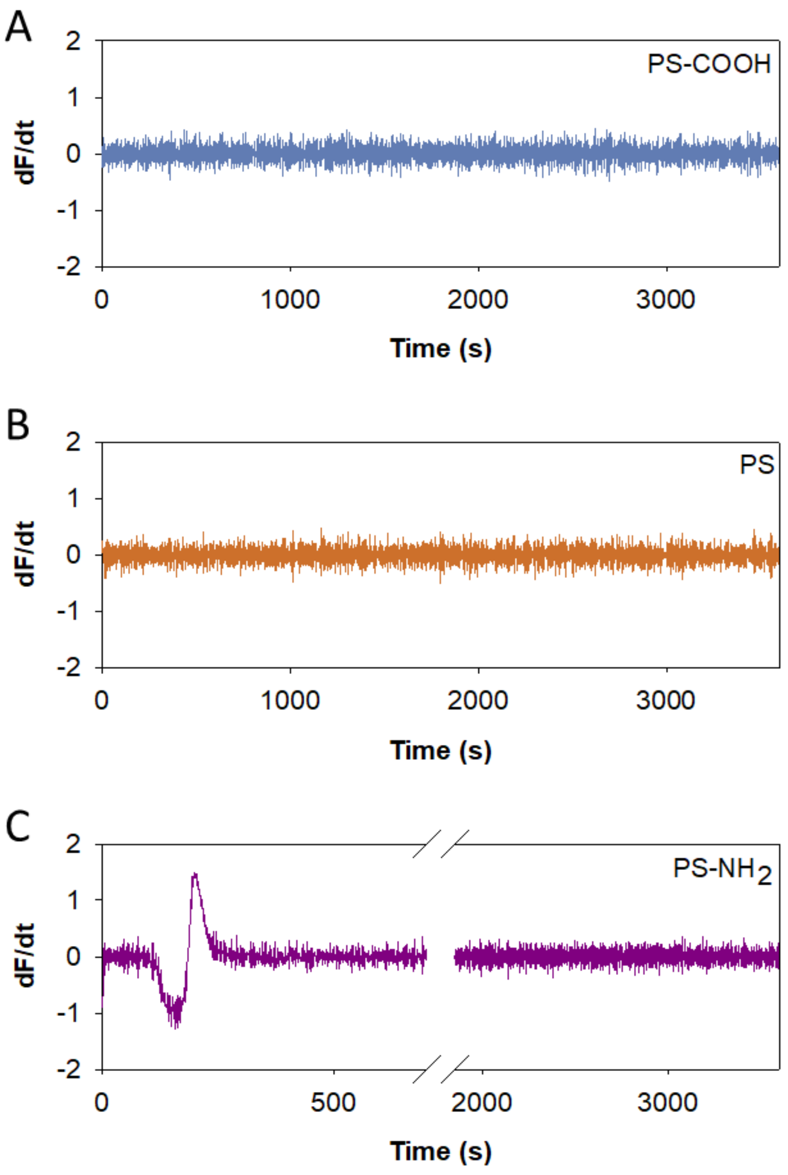

Figure S3. Time derivatives of frequency at the seventh overtone during deposition of PS-COOH (A), PS (B), PS-NH2 (C) nanoparticles suspension (in NaCl 150 mM with HEPES 5 mM pH 7.4) on 5:3:2 DOPC:DOPE:DOPS membranes.

4. Normalized frequencies and dissipation changes

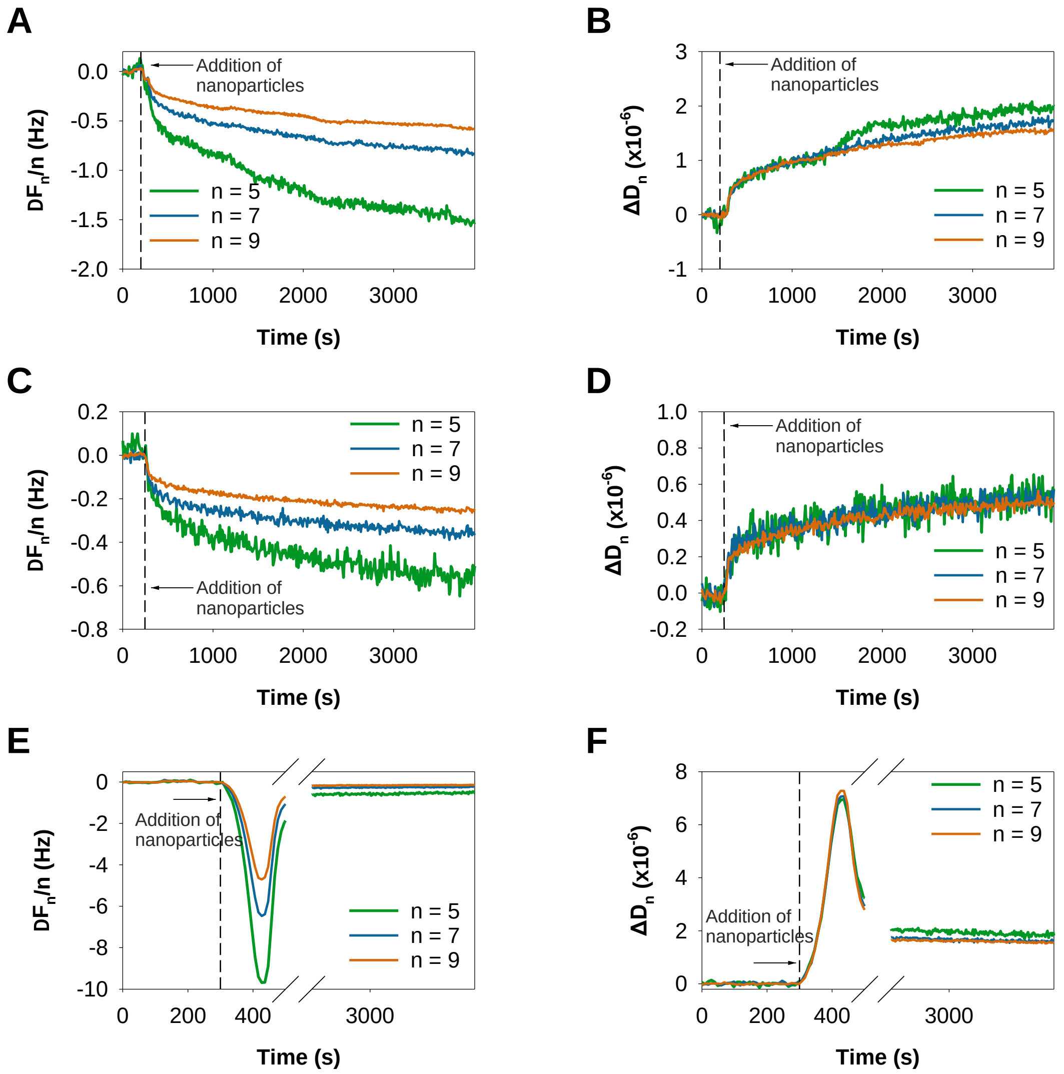

Figure S4. Representative normalized frequency (ΔFn/n) and ΔD changes for the fifth, seventh, and ninth overtones as a function of time for PS-COOH (A-B), PS (C-D) and PS-NH2 (E-F). For the case of PS-NH2, when the SLBs are exposed to these NPs, the immediate and rapid decrease in frequency is indicative of a strong adsorption of the particles to the bilayer, while the associated dissipation values suggest the existence of a soft film. Then, the continuous exposure of the SLBs to the particle suspension results in an increase frequency, associated with a mass loss. Also, the differences in normalized frequencies among the overtones are larger in the transient regime than

in the stationary one, implying that the composite layer has become more rigid. A likely cause for this confluence of signals is a remodeling of the layer due to penetration of PS-NH2 particles, inducing a re-organization of the lipid membrane and in lipid removal[1].

5. **Illustration of nanoparticles deposition**

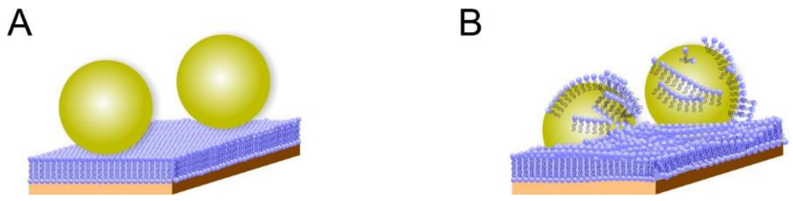

Figure S5. PS and PS-COOH nanoparticles deposition on SLB (**A**). PS-NH_2 induce lipid extraction from the SLB or the creation of water defects that induce lipid reorganizations (**B**). (Buffer NaCl 150 mM with HEPES 5 mM pH 7.4, 5:3:2 DOPC:DOPE:DOPS membrane)

6. Hydrophobicity assay with Rose Bengal

Nanoparticle hydrophobicity was determined by dye absorption using a protocol adapted from Xiao and Wiesner (2012). Briefly, 20 mg/L of Rose Bengal (RB) was added to the nanoparticle with a range of increasing surface area (~9– 18 μm^2 x10^9). Control was set by adding RB to UW to account for the absorption of RB into centrifuge tube filter. After mixing and incubation in NaCl 150 mM buffered with HEPES 5 mM at pH 7.4 (assay conditions used in QCM-D and electrophysiology) for 3h, RB partitioned between NPs surface and water. The samples were centrifugated and the free RB of the supernatant was determined by UV-visible spectroscopy (Varian Cary 300 BIO, Agilent Santa Clara, CA, USA) at a wavelength λ=543nm. The partition quotient (PQ) was calculated as follows:

$$PQ = \frac{RB_a}{RB_w},$$

where RB_a is the mass of RB adsorbed on the particle surface calculated as the difference between RB concentration in supernatant and in the particle free control, and RB_w is the mass of particle free control. The PQ is then plotted as a function of particle total surface area (TSA) obtaining a straight line for each nanoparticle. The TSA was calculated assuming that the NPs were monodispersed and using the diameter measured by DLS. The slope obtained from linear regression is considered as an indicator of the surface hydrophobicity [2–4].

7. Nanoparticles hydrophobicity results

Table S1. Nanoparticles hydrophobicity measured as slope of PQ (partition quotient) vs TSA (particle total surface area).

**Nanoparticle**	**PQ/TSA (1/ µm^2 x10^9)**	**R^2**
PS-COOH	0.0143 ± 0.0007	0.997
PS	0.028 ± 0.004	0.981
PS-NH_2	0.025 ± 0.005	0.961

8. QCM-D scheme

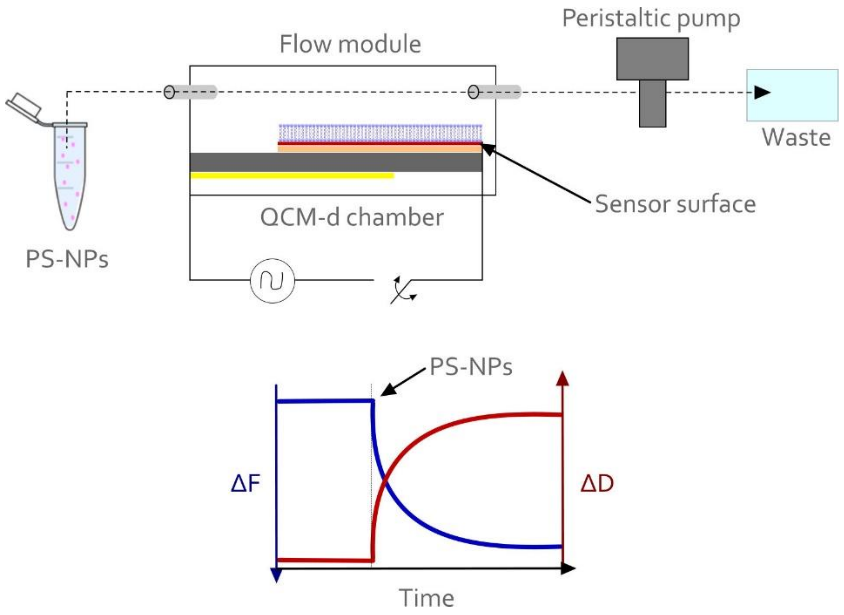

Figure S6. Illustration of QCM-D experiments. When the nanoparticles are attached to the supported lipid bilayer the resonance frequency of the crystal sensor decreases, due to the increase in mass, and the dissipation increase.

9. Planar bilayer formation

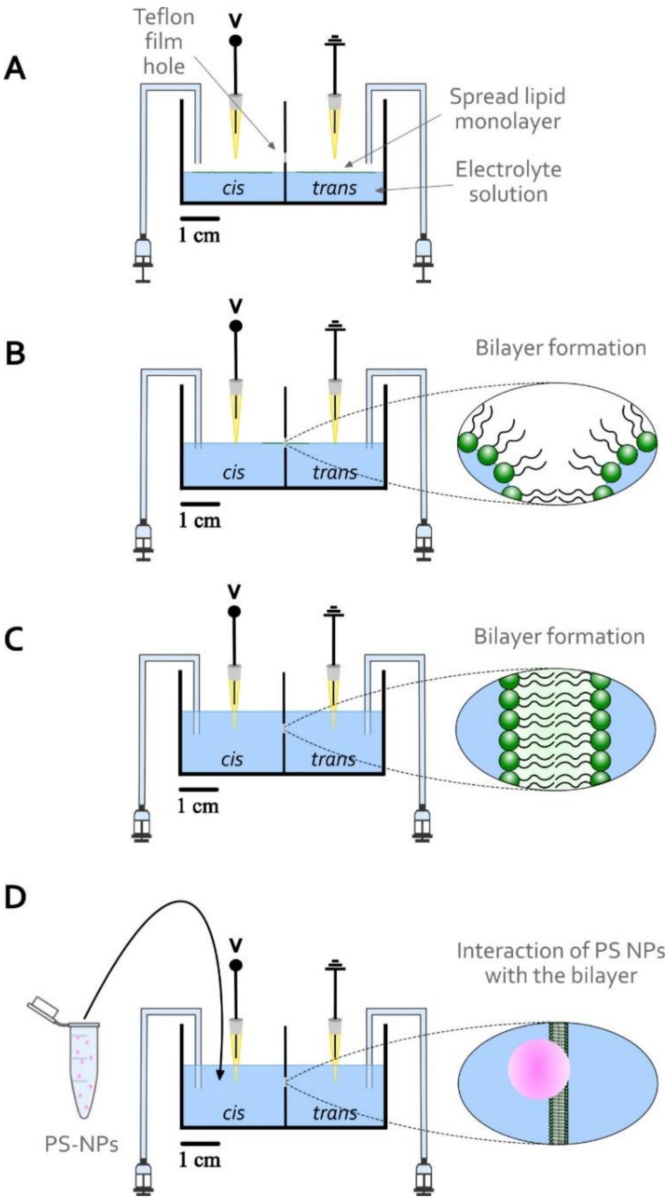

Figure S7. Formation of the lipid bilayer by apposition of two monolayers. To form the monolayer, small drops of lipid in pentane are spread on solution subphases of each compartment (**A**). Solutions level is gently raised (**B**). The solution levels completely cover the hole and the bilayer is formed by opposition of the two monolayers (**C**). Addition of nanoparticles at final concentrations of 100 ppm to the *cis* side of the chamber while *trans* electrode is set to ground (**D**).

References

(1) Bailey, C. M.; Kamaloo, E.; Waterman, K. L.; Wang, K. F.; Nagarajan, R.; Camesano, T. A. Size Dependence of Gold Nanoparticle Interactions with a Supported Lipid Bilayer: A QCM-D Study. *Biophys. Chem.* **2015**, *203–204*, 51–61. https://doi.org/10.1016/j.bpc.2015.05.006.

(2) Xiao, Y.; Wiesner, M. R. Characterization of Surface Hydrophobicity of Engineered Nanoparticles. *J. Hazard. Mater.* **2012**, *215–216*, 146–151. https://doi.org/10.1016/j.jhazmat.2012.02.043.

(3) Muller, R. H.; Davis, S. S.; Illum, L.; Mak, E. Particle Charge and Surface Hydrophobicity of Colloidal Drug Carriers. *Target. Drugs With Synth. Syst.* **1986**, 239–263. https://doi.org/10.1007/978-1-4684-5185-6_18.

(4) Mitzel, M. R.; Sand, S.; Whalen, J. K.; Tufenkji, N. Hydrophobicity of Biofilm Coatings Influences the Transport Dynamics of Polystyrene Nanoparticles in Biofilm-Coated Sand. *Water Res.* **2016**, *92* (514), 113–120. https://doi.org/10.1016/j.watres.2016.01.026.
